# The role of MRI patterns in predicting neurologic deficits in spinal ependymomas

**DOI:** 10.1007/s11060-025-05134-6

**Published:** 2025-07-15

**Authors:** Caner Sarıkaya, Cumhur Kaan Yaltırık, Eyüp Varol, Evren Yüvrük, Mehmet Reşit Önen, Sait Naderi

**Affiliations:** 1 Department of Neurosurgery, Maltepe University Hospital, İstanbul, Turkey; 2 Department of Neurosurgery, Medicana Ataköy hospital, İstanbul, Turkey; 3 Department of Neurosurgery, VM Medical Park Maltepe Hospital, İstanbul, Turkey; 4 Department of Neurosurgery, SN İstanbul Brain and Spine Center, İstanbul, Turkey

**Keywords:** Spinal ependymoma, MRI, Motor weakness, Intratumoral hemorrhage, Syrinx, Tumor volume, Longitudinal extension, Neurological outcome, Logistic regression, McCormick scale

## Abstract

**Objective:**

Spinal ependymomas are the most common intramedullary spinal cord tumors, with surgical resection being the primary treatment modality. This study aimed to evaluate the prognostic value of preoperative MRI features in predicting motor deficits in patients undergoing surgery for spinal ependymomas.

**Methods:**

We retrospectively analyzed 71 patients with histopathologically confirmed spinal ependymomas who underwent surgical resection between 2009 and 2021. Preoperative MRI features—including tumor volume, cystic components, syrinx formation, intratumoral hemorrhage, and longitudinal tumor extension—were assessed. Motor strength was recorded pre- and postoperatively. Statistical analysis included univariate comparisons and multivariate logistic regression to identify independent predictors of neurological deficits.

**Results:**

Preoperative motor weakness was significantly associated with syrinx formation (*p* = 0.012), intratumoral hemorrhage (*p* = 0.015), and greater tumor length (*p* = 0.037). Postoperative motor deterioration was significantly correlated with intratumoral hemorrhage (*p* = 0.025), increased tumor volume (*p* = 0.007), and longitudinal extension (*p* = 0.001). In multivariate analysis, the number of vertebral levels involved was the only independent predictor of postoperative motor weakness (OR = 1.737; 95% CI: 1.063–2.838; *p* = 0.028). No independent predictors were identified for preoperative weakness, although syrinx formation showed a trend toward significance.

**Conclusion:**

Preoperative MRI findings—particularly intratumoral hemorrhage, syrinx formation, and longitudinal extension—are associated with motor deficits in spinal ependymomas. However, only longitudinal tumor extension was an independent predictor of postoperative neurological deterioration. These findings highlight the importance of comprehensive radiological assessment in preoperative planning and risk stratification. Small sample size and short-term postoperative assessment represent important limitations of this study.

## Introduction

Ependymomas account for approximately 2–4% of all central nervous system (CNS) tumors and represent the most common type of intramedullary spinal tumors, comprising nearly 60% of cases [5, 16, 22]. These tumors arise from ependymal cells lining the central canal of the spinal cord and can present with a range of clinical symptoms depending on their size, location, and associated spinal cord compression. Although ependymomas are generally slow-growing, they can lead to significant neurological deficits if left untreated. Early surgical intervention is widely recommended to prevent irreversible neurological impairment and optimize functional recovery [1, 10, 14]. However, despite surgical advancements, complete tumor removal can be challenging, particularly in cases where the tumor is adherent to the spinal cord or exhibits an infiltrative growth pattern. As a result, spinal ependymomas are associated with a relatively high rate of surgical morbidity and can lead to temporary or permanent neurological deficits, even in cases of successful gross-total resection [9, 18, 21].

Given these challenges, identifying prognostic factors that influence postoperative neurological outcomes is of critical importance. Several studies have investigated factors such as patient demographics, duration of symptoms before surgery, preoperative neurological deficits, extent of tumor resection, tumor pathology (World Health Organization [WHO] grading), tumor size, and tumor location in predicting postoperative functional status [1, 2, 26]. The presence of preoperative neurological deficits, in particular, has been strongly correlated with poorer postoperative outcomes [3, 4, 6–8, 10, 13, 24]. However, the impact of specific radiological tumor characteristics on surgical prognosis remains an area of active investigation.

Magnetic resonance imaging (MRI) is the gold standard for diagnosing and characterizing spinal ependymomas. Preoperative MRI findings provide crucial information regarding tumor size, margins, and internal composition, including the presence of cystic changes, syrinx formation, and intratumoral hemorrhage. Previous studies have suggested that these radiological features may play a role in predicting neurological outcomes following surgical resection. For instance, syrinx formation has been associated with a more favorable surgical prognosis, as it may provide a natural cleavage plane between the tumor and the spinal cord, facilitating safer resection [23]. Conversely, the presence of intratumoral hemorrhage may indicate a more aggressive tumor biology and has been linked to a higher likelihood of postoperative neurological deficits [4, 19].

Despite these findings, the prognostic significance of MRI characteristics in spinal ependymomas remains incompletely understood. In this study, we aimed to retrospectively evaluate the relationship between preoperative MRI findings and postoperative neurological deficits in patients undergoing surgical resection for spinal ependymomas. Specifically, we analyzed the impact of tumor volume, cystic components, syrinx formation, intratumoral hemorrhage, and tumor longitudinal extension on functional outcomes. By identifying key radiological predictors of postoperative deficits, this study seeks to contribute to the growing body of literature on spinal ependymomas and aid in preoperative risk stratification and surgical decision-making.

## Materials and methods

### Study design and patient selection

This retrospective observational study was conducted at the Neurosurgery Department of Istanbul Ümraniye Training and Research Hospital, following approval from the Institutional Ethics Committee (Decision No: B.10.1.TKH.4.34.H.GP.0.01/49). Medical records from 287 patients who underwent spinal tumor surgery with intraoperative neuromonitoring between 2009 and 2021 were screened. A total of 71 patients with histopathologically confirmed spinal ependymomas and complete clinical and imaging data were included in the final analysis.

### Inclusion criteria

Patients were eligible for inclusion if they met the following conditions:

- Underwent surgical resection for a histopathologically confirmed spinal ependymoma,
- Had available preoperative and postoperative MRI scans,
- Possessed complete clinical documentation, including neurological assessments,
- Surgery was performed under intraoperative neuromonitoring.

### Exclusion criteria

Patients were excluded from the study based on the following:

- Absence of histological confirmation of spinal ependymoma,
- Incomplete radiological or clinical documentation,
- Prior spinal surgery or radiation therapy,
- Presence of other spinal pathologies such as metastases, infections, or demyelinating diseases.

### Radiological evaluation

**Fig. 1:**
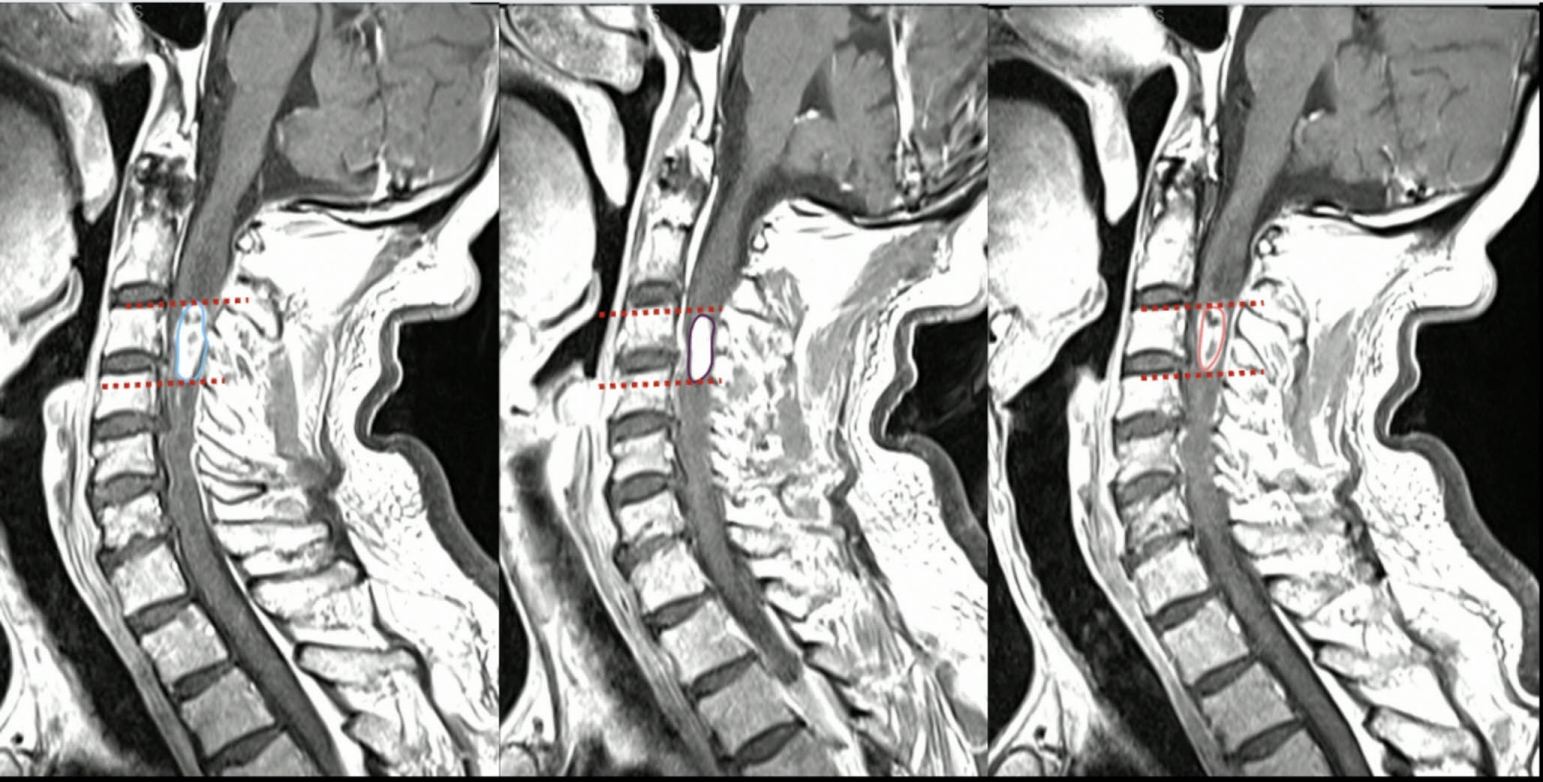
Sagittal T2-weighted MRI images of the cervical spine showing three consecutive slices from the same patient. An intradural extramedullary tumor is visible in all three slices. Tumor tissue was manually segmented and annotated (outlined in red). The lesion was confined to a single vertebral segment in cranio-caudal extension

**Fig. 2:**
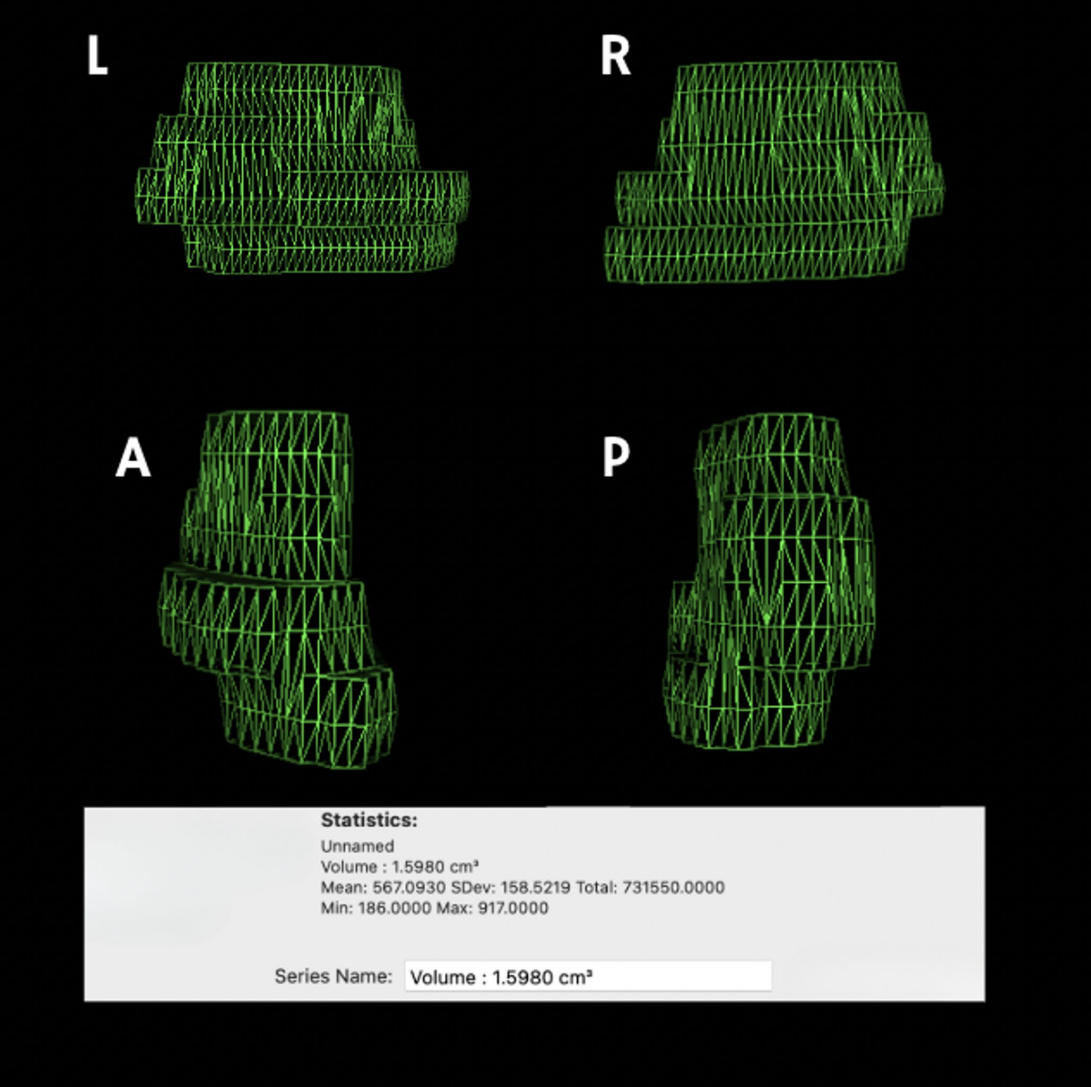
3D volumetric reconstruction of the segmented intradural extramedullary tumor from the same case, performed using Horos software. Tumor tissue was manually labeled, and the total tumor volume was calculated as 1.5980 cm³. Views from left (**L**), right (**R**), anterior (**A**), and posterior (**P**) aspects are shown

All patients underwent preoperative and early postoperative (< 24 h) MRI using a 1.5 Tesla scanner. The imaging protocol included sagittal and axial T1-weighted sequences with contrast, and T2-weighted sequences. MRI features were evaluated independently by two experienced neuroradiologists using Horos^®^ MD software (Fig. 1 and Fig. 2). The following parameters were analyzed:

- **Tumor Volume**: Volumetric analysis was performed through manual segmentation of the tumor on serial axial slices.
- **Cystic Component**: Defined as intratumoral fluid-containing areas distinct from syrinxes.
- **Syrinx Formation**: Defined as a non-neoplastic, fluid-filled cavity within the spinal cord adjacent to the tumor.
- **Intratumoral Hemorrhage**: Identified as T1 hyperintensity or heterogeneous signal intensity on T2-weighted images.
- **Longitudinal Extension**: Recorded both as the number of vertebral segments involved and the cranio-caudal length of the tumor in millimeters.
- **Tumor Localization**: Categorized anatomically into craniovertebral junction, cervical, thoracic, lumbar, and sacral regions.

### Clinical and surgical assessment

All surgeries were performed with the goal of gross-total resection whenever feasible. Intraoperative neurophysiological monitoring, including somatosensory evoked potentials (SSEPs) and motor evoked potentials (MEPs), was utilised in all cases. No reoperations occurred within the follow-up period.

Postoperative motor function was evaluated within 24 h after surgery and at hospital discharge. Long-term functional outcomes were not assessed in this study. Neurological status was evaluated using the modified McCormick functional grading system and detailed motor strength examination. Preoperative and postoperative grades and motor function were compared. Surgical approaches included total laminectomy, laminoplasty, and hemilaminectomy, performed by experienced neurosurgeons using microsurgical techniques and intraoperative neurophysiological monitoring.

### Statistical analysis

All statistical analyses were performed using IBM SPSS Statistics version 27 (IBM Corp., Armonk, NY, USA). The distribution of continuous variables was assessed via descriptive methods (skewness, kurtosis, and standard deviation/mean ratio), graphical inspection (Q-Q plots and histograms), and the Kolmogorov–Smirnov test.

Categorical variables were expressed as frequency and percentage (n, %), and continuous variables were summarized as mean ± standard deviation (SD) or median and interquartile range (IQR), depending on distribution.

For comparisons between two groups, the independent samples t-test was used for normally distributed variables, while the Mann–Whitney U test was applied for non-normally distributed variables. Categorical variables were compared using Fisher’s exact test.

Multivariate binary logistic regression analysis was conducted to identify independent predictors of preoperative and postoperative motor weakness. The strength of association was expressed as odds ratios (OR) with 95% confidence intervals (CI). Model fit was evaluated using the Hosmer–Lemeshow test, and explanatory power was reported via Nagelkerke R². A *p*-value < 0.05 (two-tailed) was considered statistically significant.

In univariate analysis, variables exhibiting notable correlations (*p* < 0.1) were included in the multivariate logistic regression model to find independent predictors. This covered tumour volume, intratumoral bleeding, syrinx development, and longitudinal tumour extension.

## Results

### Patient characteristics

A total of 71 patients who underwent surgical resection of histologically confirmed spinal ependymomas were included in the study. The mean age of the cohort was 42.68 ± 13.6 years (range: 14–74), and 41 patients (57.7%) were female. The most common pathological subtype was classic ependymoma (56.3%), followed by myxopapillary (32.4%), tanycytic (7%), subependymoma (2.8%), and anaplastic ependymoma (1.4%). According to the WHO classification, 25 patients (35.2%) were Grade I, 45 (63.4%) Grade II, and one patient (1.4%) was Grade III.

Anatomically, tumors were most frequently located in the lumbar spine (45.1%), followed by the cervical (32.4%) and thoracic (14.1%) regions. The craniovertebral junction and sacral spine each accounted for 4.2% of cases. The median number of vertebral levels involved was 2 (IQR: 1–3), while the median craniocaudal tumor extension was 23 mm (IQR: 11–43). Mean tumor diameter was 11.29 ± 5.23 mm, and median tumor volume was 1.23 cm³ (IQR: 0.36–3.24). Preoperative MRI demonstrated cystic components in 38 cases (53.5%), syrinx formation in 23 cases (32.4%), and intratumoral hemorrhage in 24 cases (33.8%). Detailed demographic and radiological characteristics are provided in Table 1.

**Table 1:** Patient characteristics

Variable	Statistic
**Age(year)**, ***mean ± SD(range)***	42.68 ± 1.60 (14–74)
**Gender**, ***n(%)***	
Female	41(57.7)
Male	30(42.3)
**Pathology**, ***n(%)***	
Ependymoma	40(56.3)
Myxopapillary Ependymoma	23(32.4)
Tanycytic Ependymoma	5(7)
Subependymoma	2(2.8)
Anaplastic Ependymoma	1(1.4)
**Grade**, ***n(%)***	
I	25(35.2)
II	45(63.4)
III	1(1.4)
**Vulume**, ***median(IQR)***	1.23(0.36–3.24)
**Cyst**, ***n(%)***	38(53.5)
**Syrinx**, ***n(%)***	23(32.4)
**Bleeding site**, ***n(%)***	24(33.8)
**Tumor localization**, ***n(%)***	
CVB	3(4.2)
Cervical	23(32.4)
Thoracic	10(14.1)
Lumbar	32(45.1)
Sacrum	3(4.2)
**Number of vertebrae levels the tumor extended**, ***median(IQR)***	2(1–3)
**Tumor extension** [19], ***median(IQR)***	23(11–43)
**Tumor size** [19], ***mean ± SD***	11.29 ± 5.23

**SD**, Standard deviation; **IQR**, Inter Quantile Range

### Preoperative neurological deficits and radiological correlation

Preoperative motor weakness was observed in 6 patients (8.5%). Syrinx formation and intratumoral hemorrhage were both significantly associated with preoperative motor weakness. Patients with syrinx had a higher rate of weakness compared to those without (21.7% vs. 2.1%, *p* = 0.012), as did those with hemorrhage (20.8% vs. 2.1%, *p* = 0.015). Additionally, patients with preoperative weakness had significantly more extensive longitudinal tumor involvement (median: 4 vertebral levels vs. 2; *p* = 0.037). While tumor volume was higher in the weakness group (median: 2.24 cm³ vs. 1.03 cm³), this did not reach statistical significance (*p* = 0.154). No significant associations were found between preoperative weakness and age, gender, tumor location, WHO grade, or cystic changes (Table 1).

### Postoperative motor deficits and imaging predictors

New or worsened postoperative motor weakness was observed in 10 patients (14.1%). The presence of intratumoral hemorrhage was significantly associated with postoperative deficits (29.2% vs. 6.4%; *p* = 0.025). Similarly, patients with postoperative weakness had significantly larger tumor volumes (median: 5.51 cm³ vs. 0.69 cm³; *p* = 0.007) and more extensive vertebral involvement (median: 4 vs. 2 vertebral levels; *p* = 0.001). Although syrinx presence did not reach statistical significance in this context (*p* = 0.718), the trend remained. Dichotomized analysis further supported that involvement of two or more vertebral levels increased the risk of postoperative weakness (22.5% vs. 3.2%, *p* = 0.035). No significant associations were found for tumor location, WHO grade, tumor diameter, or total tumor extension in millimeters (Table 2).

**Table 2:** Preoperative and postoperative motor weakness rates according to diagnostic characteristics of patients

Variable		Motor Weakness					
	*n*	Pre-operative		*P*-value	Post-operative		*P*-value
		No (*n* = 65)	Yes (*n* = 6)		No (*n* = 61)	Yes (*n* = 10)	
**Age(year)**, ***mean ± SD***	71	42.22 ± 13.03	47.67 ± 18.64	0.348^a^	43.28 ± 13.31	39.00 ± 14.82	0.357^a^
**Gender**, ***n(%)***				0.233^c^			0.733^c^
Female	41	39(95.1)	2(4.9)		36(87.8)	5(12.2)	
Male	30	26(86.7)	4(13.3)		25(83.3)	5(16.7)	
**Pathology**, ***n(%)***				0.611^c^			0.337^c^
Ependymoma	40	37(92.5)	3(7.5)		36(90)	4(10)	
Myxopapillary Ependymoma	23	21(91.3)	2(8.7)		19(82.6)	4(17.4)	
Tanycytic Ependymoma	5	4(80)	1(20)		4(80)	1(20)	
Subependymoma	2	2(100)	0(0)		1(50)	1(50)	
Anaplastic Ependymoma	1	1(100)	0(0)		1(100)	0(0)	
**Grade**, ***n(%)***				> 0.999^c^			0.307^c^
I	25	23(92)	2(8)		20(80)	5(20)	
II-III	45	42(91.3)	4(8.7)		41(89.1)	5(10.9)	
**Vulume**, ***median(IQR)***	71	1.03 (0.35–3.22)	2.24 (1.26–8.73)	0.154^b^	0.69 (0.31–2.76)	5.51 (2.19–12.86)	**0.007** ^**b**^ *
**Cyst**, ***n(%)***				0.206^c^			0.320^c^
No	33	32(97)	1(3)		30(90.9)	3(9.1)	
Yes	38	33(86.8)	5(13.2)		31(81.6)	7(18.4)	
**Syrinx**, ***n(%)***				**0.012** ^**c**^ *			0.718^c^
No	48	47(97.9)	1(2.1)		42(87.5)	6(12.5)	
Yes	23	18(78.3)	5(21.7)		19(82.6)	4(17.4)	
**Bleeding site**, ***n(%)***				**0.015** ^**c**^ *			**0.025** ^**c**^ *
No	47	46(97.9)	1(2.1)		44(93.6)	3(6.4)	
Yes	24	19(79.2)	5(20.8)		17(70.8)	7(29.2)	
**Tumor localization**, ***n(%)***				0.530^c^			0.619^c^
CVB	3	2(66.7)	1(33.3)		2(66.7)	1(33.3)	
Cervical	23	21(91.3)	2(8.7)		21(91.3)	2(8.7)	
Thoracic	10	9(90)	1(10)		8(80)	2(20)	
Lumbar	32	30(93.8)	2(6.3)		27(84.4)	5(15.6)	
Sacrum	3	3(100)	0(0)		3(100)	0(0)	
**Number of vertebrae levels the tumor extended**, ***median(IQR)***	71	2(1–2)	4(2–5)	**0.037** ^**b**^ *	2(1–2)	4(2–6)	**0**,**001**^b^*
1	31	30(96.8)	1(3.2)	0.222^c^	30(96.8)	1(3.2)	**0.035** ^**c**^ *
≥ 2	40	35(87.5)	5(12.5)		31(77.5)	9(22.5)	
**Tumor extension** [19], ***median(IQR)***	71	23(11–42)	28(9–52)	0.934^b^	21(10–39)	39(12–54)	0.237^b^
**Tumor size** [19], ***mean ± SD***	71	11.26 ± 5.21	11.67 ± 5.85	0.856^a^	11.34 ± 5.23	11.01 ± 5.46	0.855^a^

*p < 0.05; **a**, Independent samples t-test; **b**, Mann-Whitney U test; **c**, Fisher’s Exact Test

### Multivariate logistic regression analysis

Multivariate logistic regression analysis was conducted to identify independent predictors of both preoperative and postoperative motor weakness. For postoperative weakness, longitudinal tumor involvement (number of vertebrae) emerged as the only independent risk factor (OR = 1.737; 95% CI: 1.063–2.838; *p* = 0.028). Tumor volume and intratumoral hemorrhage were not significant in the adjusted model. This model showed adequate calibration (Hosmer–Lemeshow *p* = 0.155) and accounted for 30.5% of the variance (Nagelkerke R² = 0.305).

For preoperative weakness, none of the variables reached statistical significance in the multivariate model. Syrinx formation showed a trend toward significance (OR = 7.76; 95% CI: 0.726–82.971; *p* = 0.090), while intratumoral hemorrhage and tumor length were not significant predictors. The model explained approximately 33% of the variance (Nagelkerke R² = 0.329), but the small number of preoperative deficit cases likely limited its power (Tables 3).

**Table 3:** Results of multivariate logistic regression analysis

Dependent variable, Post-operative Weakness (1 = Yes, No = 0)					
Variable	B	SE	OR (95% CI)	*P*-value	Mdel summary
Vulume	0.012	0.043	1.012(0.931-1.100)	0.773	χ²=13.202; *p* = 0.004
Bleeding site	0.763	0.914	2.145(0.358–12.872)	0.404	Nagelkerke R^2^ = 0.305
Number of vertebrae levels the tumor extended	0.552	0.251	1.737(1.063–2.838)	0.028*	H&L Test. *p* = 0.155
Constant	-3.771	0.796	0.023	< 0.001	

**Dependent variable**,** Pre-operative Weakness*(1 = Yes***,*** No = 0)***					
**Variable**	**B**	**SE**	**OR (95% CI)**	***P*** **-value**	**Mdel summary**
Syrinx	2.049	1.209	7.760(0.726–82.971)	0.090	χ²=11.082; *p* = 0.011
Bleeding site	1.388	1.322	4.007(0.300-53.466)	0.294	Nagelkerke R^2^ = 0.329
Number of vertebrae levels the tumor extended	0.232	0.267	1.261(0.747–2.127)	0.385	H&L Test. *p* = 0.074
Constant	-5.002	1.370	0.007	< 0.001	

**OR**, odds ratio, **B**, Regression estimation, **CI**, Confidence interval; **SE**, Standard error; **H&L**, Hosmer and Lemeshow Test

## Discussion

The prognosis of spinal ependymomas is influenced by tumor-related factors, surgical complexity, and preoperative neurological condition. In this study, we investigated the relationship between specific MRI characteristics and neurological deficits, focusing on the predictive role of radiological features such as tumor volume, hemorrhage, syrinx formation, and longitudinal extension. Our findings suggest that among these, longitudinal tumor extension is the most reliable independent predictor of postoperative motor weakness.

Preoperative motor weakness, observed in 8.5% of patients, was significantly associated with syrinx formation, intratumoral hemorrhage, and increased vertebral involvement in univariate analysis. These results are consistent with prior reports that identified preoperative neurological status and tumor size as important prognostic indicators in spinal intramedullary tumors [1, 10–12, 21, 25]. However, in multivariate logistic regression, no variable retained statistical significance, although syrinx formation demonstrated a strong trend (*p* = 0.090), indicating a potential association worth further investigation in larger cohorts.

Postoperative neurological deterioration, found in 14.1% of patients, was significantly associated with hemorrhagic tumors, greater tumor volume, and more extensive longitudinal extension. In multivariate analysis, only longitudinal extension remained independently significant (OR = 1.737, *p* = 0.028), indicating that the length of the tumor is a stronger predictor of postoperative outcome than either volume or hemorrhage. This is in line with earlier findings that tumors involving ≥ 3 vertebral segments increase the risk of postoperative neurological morbidity due to more extensive spinal cord manipulation [4, 19]. The presence of syrinx, previously considered a favorable factor due to the potential for a natural dissection plane, was instead significantly associated with preoperative motor weakness in our study. However, its lack of association with postoperative outcomes suggests that it may be more reflective of prior spinal cord damage than a predictor of surgical success [23].

Tumor volume, although significantly associated with postoperative motor deficits in univariate analysis (*p* = 0.007), did not maintain this association in the multivariate regression model. This finding suggests that tumor volume, while reflecting overall mass effect, may be secondary in prognostic importance when adjusted for other structural features, particularly longitudinal extension. It is likely that larger tumor volumes correlate with greater cranio-caudal tumor spread, leading to multicollinearity within the regression model and diminishing the independent predictive power of volume. This interpretation is consistent with previous studies that have highlighted the interdependence between volumetric burden and tumor length [4, 19].

Previous reports have attempted to define critical tumor volume thresholds that may predict neurological deterioration. For example, Ozkan et al. proposed a scoring system incorporating tumor volume as one of the prognostic components [19], while Ma et al. suggested that volumes exceeding 2.5 cm³ are associated with worse functional outcomes [15]. However, these thresholds often vary between cohorts and imaging protocols, which limits their universal applicability. In our study, despite observing a significant association between tumor volume and postoperative weakness in unadjusted analyses, we found that longitudinal tumor extension was a more consistent and reproducible parameter, likely due to its anatomical relevance to surgical planning. Correlation analysis revealed a moderate relationship between tumour volume and longitudinal extension (Spearman’s ρ = XX, *p* < 0.05), suggesting potential multicollinearity. Variance inflation factor (VIF) analysis demonstrated acceptable values (VIF < X), supporting the inclusion of longitudinal extension as a more anatomically interpretable predictor in multivariate analysis.

Histological tumor grade, pathological subtype, and anatomical localization did not significantly correlate with pre- or postoperative motor weakness in our patient population. These findings align with prior reports suggesting that radiological features may offer superior prognostic value compared to histopathological classification [3, 8, 10, 13]. For instance, Karikari et al. reported that tumor histology was not predictive of functional outcomes, especially in cases where gross total resection was achieved [8], and Domazet et al. similarly emphasized that imaging features and preoperative neurological status were stronger prognostic indicators than tumor grade [3]. Likewise, Li et al. found that long-term outcomes in spinal ependymomas were more closely associated with tumor size and neurological presentation than with histopathological subtype [13].

Additionally, our findings resonate with the observations of Peker et al., who demonstrated that tumors with greater longitudinal spread were more likely to be associated with postoperative dysesthesia, likely due to increased manipulation and traction of the spinal cord during microsurgical dissection [20]. Our findings align with recent multicenter data, such as that of Naito et al., which emphasise the prognostic significance of tumour length and preoperative neurological status. Furthermore, while scoring systems like the SOURSE score have incorporated volume metrics, our results suggest that longitudinal extension may offer a simpler, reproducible alternative for surgical risk stratification [17]. Although our study did not directly assess postoperative sensory changes, the significant relationship between greater vertebral involvement and motor decline supports the broader conclusion that longitudinal extension is a critical parameter for anticipating surgical morbidity. Identifying longitudinal tumour extension as an independent predictor can aid surgeons in preoperative counselling by highlighting cases that may carry higher surgical risk due to extensive spinal cord manipulation. This may influence surgical approach decisions, intraoperative neuromonitoring strategies, and postoperative care planning.

Despite its valuable findings, our study has several limitations. First, its retrospective nature introduces potential biases related to data collection and patient selection. A prospective, multi-center study with standardized assessment protocols would be needed to validate our results. Second, the relatively small sample size (*n* = 71) limits the generalizability of our findings, particularly in subgroup analyses of different tumor grades and histological subtypes. The limited number of patients with preoperative (*n* = 6) and postoperative (*n* = 10) motor deficits restricts the statistical power of the logistic regression analyses and may increase the risk of model overfitting, as reflected by wide confidence intervals. Third, while we identified significant MRI predictors of neurological deficits, we did not assess other factors such as intraoperative findings, tumor resection extent, or long-term functional recovery beyond the postoperative period. The short-term assessment of postoperative motor function limits the evaluation of potential neurological recovery or delayed deterioration. Longer follow-up would provide a more comprehensive picture of functional outcomes. Future studies should incorporate these variables to provide a more comprehensive analysis of prognostic factors. Finally, we did not evaluate molecular markers, which have been increasingly recognized as prognostic indicators in CNS tumors, including ependymomas [22, 26].

## Conclusion

This study highlights the prognostic relevance of specific MRI features in spinal ependymomas, with a particular emphasis on the role of longitudinal tumor extension. While intratumoral hemorrhage, syrinx formation, and tumor volume were significantly associated with motor deficits in univariate analyses, only longitudinal extension remained an independent predictor of postoperative motor weakness in multivariate logistic regression. These findings suggest that tumor length, as reflected by the number of vertebral levels involved, is a robust and reproducible parameter for preoperative risk stratification.

The lack of significant correlation between neurological outcomes and histological grade or tumor location further supports the clinical utility of imaging-based markers over traditional pathological classification in surgical planning. Given the potential for irreversible neurological deficits, particularly in tumors with extensive craniocaudal spread, early recognition of high-risk imaging features is essential for informed surgical decision-making.

## Data Availability

No datasets were generated or analysed during the current study.
